# Comparative Torsional Properties via Numerical Simulation of Triply Periodic Minimal Surfaces (TPMS): Diamond, Gyroid and Primitive Structures

**DOI:** 10.3390/polym18060736

**Published:** 2026-03-18

**Authors:** Mikhail Skibar, Rahmat Agung Susantyoko, Salman Pervaiz

**Affiliations:** 1 DEWA R&D Centre, Dubai Electricity and Water Authority, Dubai P.O. Box 564, United Arab Emiratesrahmat.susantyoko@dewa.gov.ae (R.A.S.); 2 Department of Mechanical and Industrial Engineering, Rochester Institute of Technology—Dubai Campus, Dubai P.O. Box 341055, United Arab Emirates; 3 Materials and Advanced Manufacturing Research Center (MAMRC), Rochester Institute of Technology—Dubai Campus, Dubai P.O. Box 341055, United Arab Emirates

**Keywords:** TPMS, torsional, shear yield stress, gyroid, cell size

## Abstract

This work examines the simulation-based torsion properties of TPMS structures. Although TPMS structures are gaining more interest in research and potential practical applications, their torsion properties are not widely studied. In this work, sheet-based Diamond, Gyroid, and Primitive TPMS structures are analyzed numerically using the finite element method. The samples have a diameter of 20 mm and a length of 40 mm. Relative densities are 30%, 50%, and 70%, while unit cell sizes are 10 mm, 15 mm, and 20 mm. Cell geometry did not significantly affect the properties for samples with a 10 mm unit cell size. For other unit cell sizes, the shear modulus and shear yield stress were 1.5–4 times higher for the Primitive structure than for other geometries. With increasing relative density, the shear modulus and shear yield stress increased by 1.5–2 times for the Diamond and Gyroid structures, as well as for the Primitive structure with a 10 mm unit cell size. The Primitive structure with 15 mm and 20 mm unit cell sizes showed a decrease in properties with increasing relative density. Regarding the effect of unit cell size, the shear modulus and shear yield stress showed insignificant differences for the Diamond and Gyroid structures, while the Primitive structure showed dependence on unit cell size. Samples with a 15 mm unit cell size had 1.5–2 times higher shear modulus and 1.5–3 times higher shear yield stress than samples with a 10 mm unit cell size. Samples with a 20 mm unit cell size exhibited slightly lower shear modulus and shear yield stress than those with 15 mm unit cells.

## 1. Introduction

Enhancing the performance of materials has always been a fundamental goal in research and engineering. With the development of new technologies, new materials and structures continue to emerge. Lattice structures composed of multiple patterns repeated in three directions have gained considerable attention in both research and practical applications. They possess high energy absorption, allow the creation of lightweight structures, and offer enhanced fatigue properties. One possible form of these structures is the Triply Periodic Minimal Surface (TPMS). These surfaces consist of repeatable patterns without self-intersections and possess zero mean curvature at every point. They were first described by Professor Schwarz in 1865 [1]. In 1970, the National Aeronautics and Space Administration (NASA) published a report on TPMS [2]. Due to their complex shapes, the production of structures based on these surfaces was initially challenging. However, with the advent of additive manufacturing, their fabrication became feasible. Structures based on TPMS surfaces have potential applications in various areas. In water desalination, they are used as 3D-printed feed spacers for flux enhancement and biofouling mitigation in reverse osmosis and ultrafiltration processes [3]. In the biomedical field, they are applied in bone regeneration [4]. In aerospace, they can be used as lightweight structures and for heat exchange, owing to their high surface-to-volume ratio [5]. In the automotive sector, they can serve as shock-absorbing structures due to their high energy absorption capabilities. Another application in the automotive field is in airless tires [6].

Li et al. [7] focused on studying the bending performance of Diamond TPMS structures. An increase in relative density was shown to improve bending performance. With an increase in punch diameter, the energy absorption characteristics were enhanced. The properties of TPMS structures under compression have been widely studied. For example, Peng et al. [8] researched the compression and buckling properties of the Primitive TPMS structure, depending on its relative density, unit cell size, and printing direction. Reshadinezhad et al. [9] studied Diamond, Gyroid, and Primitive structures with porosity levels between 45% and 80% under compression, both numerically and experimentally. The samples were printed from Ti6Al4V titanium alloy. Simulations were performed in Abaqus, with the bottom edge fixed and a force applied to the top of the sample. A C3D10 tetrahedral mesh was utilized. For higher porosity, the Primitive structure exhibited the highest elastic modulus, yield strength, and ultimate strength, whereas the Diamond structure showed the highest values at low porosity levels. Additionally, bone implant samples were tested under compression–shear loading. Kumar et al. [10] researched Diamond and Gyroid TPMS structures under compression, fatigue, and compression–torsion. The samples were printed from titanium and experimentally tested in compression. The Gyroid and Diamond structures demonstrated similar mean stiffness, with the Diamond showing a slightly higher value. The Diamond structure also had a significantly higher yield load value than the Gyroid. Furthermore, continuous carbon fiber reinforcement has been studied. Susantyoko et al. [11] investigated the compressive mechanical properties of a Gyroid structure with continuous carbon fiber reinforcement.

Polylactic Acid (PLA) is a material widely used in additive manufacturing. TPMS structures printed from PLA have been studied in multiple works. Razi et al. [12] studied the optimization of printing speed, relative density, and unit cell size on the compression properties of PLA-based Gyroid structures. Dadashi et al. [13] investigated the skeletal Primitive structure under compression. Di Frisco et al. [14] researched the properties of hybrid TPMS structure sheet-based Primitive combined with different infills such as skeletal Gyroid, Primitive, and IWP. Spece et al. [15] investigated TPMS structures made of polyetheretherketone (PEEK). Overall, numerous studies have focused on the compression properties of TPMS structures. Torsion properties of TPMS structures may also be useful in various applications. However, only a few works have examined TPMS structures under torsion, most of which concentrate on biomedical applications for bone regeneration. For example, Yánez et al. [16] studied experimentally and numerically the torsion properties of skeletal Gyroid structures with different relative densities, in both normal and deformed arrangements, where unit cells were extended along the sample axis. Yánez et al. [17] examined the compression, torsion, three-point bending, and permeability properties of Gyroid structures, both sheet and skeletal forms, normal and modified as well as stochastic structures. All samples in these studies were printed from Ti6Al4V titanium alloy. Timercan et al. [18] analyzed the torsion properties of Gyroid and Diamond structures with various relative densities, both numerically and experimentally, using Ti6Al4V titanium alloy. Hailu et al. [19] studied Diamond and Vertically Inclined structures graded in axial and radial directions and printed from nylon. Hao et al. [20] investigated the compression and torsion behavior of concrete Primitive and Gyroid structures, including main skeletal and secondary skeletal configurations. Hailu et al. [21] examined the torsion properties of Gyroid and Primitive TPMS structures, as well as strut-based Vertically Inclined structures. Jijun Jiang et al. [22] investigated the torsion properties of Diamond, Gyroid, IWP, and Primitive structures.

To sum up, it is rare to find studies researching compression properties of TPMS structures. Although torsion properties of TPMS structures are investigated in several studies, there are still gaps in research. Most of the works investigate Gyroid structures, printed of Ti6Al4V titanium alloy, intended for biomedical applications. However, some other materials like PEEK and TPMS structures like Diamond, IWP and Primitive or lattice structure arrangements, like vertical inclined or stochastic are present in research. There are two studies that compare properties of Primitive, Gyroid and Diamond [21,22]. There are no studies found that concentrate on torsion properties of TPMS structures made of PLA. There are studies that investigate the combination of parameters like cell geometry, relative density, and unit cell size on torsional properties of TPMS structures, but the combination of all the parameters is rarely investigated. Also, there is potential in the research of different cell geometries. This study aims to fill those gaps.

## 2. Methodology

### 2.1. Design of Experiment

In this work, a full-factorial design of experiments is employed. The samples have a diameter of 20 mm and a length of 40 mm and are made of PLA material. The cell geometries considered are Diamond, Gyroid, and Primitive. These structures are widely used in research and practical applications. However, as identified in the literature review, only a few studies compare their torsion properties. The samples have relative densities of 30%, 50%, and 70%. Relative density represents the percentage of the structure’s volume compared to that of a solid cylinder. In the reviewed literature, the term porosity is often used instead; this parameter indicates the percentage of empty space within the structure and can be calculated as 100% minus the relative density (in percent). The relative densities selected for the current work are close to those in the study by Timercan et al. [18], where relative densities of 50% and 20% (or porosity levels of 50% and 80%) were used. Yánez et al. [16] examined relative densities of 25% and 10%; however, their samples were made of titanium, which is stronger than PLA. The unit cell sizes used are 10 mm, 15 mm, and 20 mm, chosen based on computational feasibility. In total, all 27 possible combinations are tested. All the samples investigated in this study are presented in Table 1.

### 2.2. Mesh Convergence Study

For every unit cell size, mesh convergence research has been done to identify the optimal mesh size. A simulation was run with different mesh sizes and the error between the maximum magnitude of the reaction moment was checked. Mesh convergence was done on a Primitive cell geometry with 30% relative density. The smallest relative density is used to check if the mesh can capture the smallest shapes in the TPMS structure. However, to increase calculation time, for larger unit cell sizes, smaller mesh densities are used, if the error stays low. The result of the mesh convergence study is presented in Table 2.

An error of less than 0.75% was considered acceptable, based on the literature review [6]. According to this, a mesh density of 45 was chosen for samples with unit cell size of 20 mm and 15 mm, and for the ones with 10 mm unit cell size, mesh density of 60 has been chosen.

### 2.3. Model Preparation

**Figure 1:**
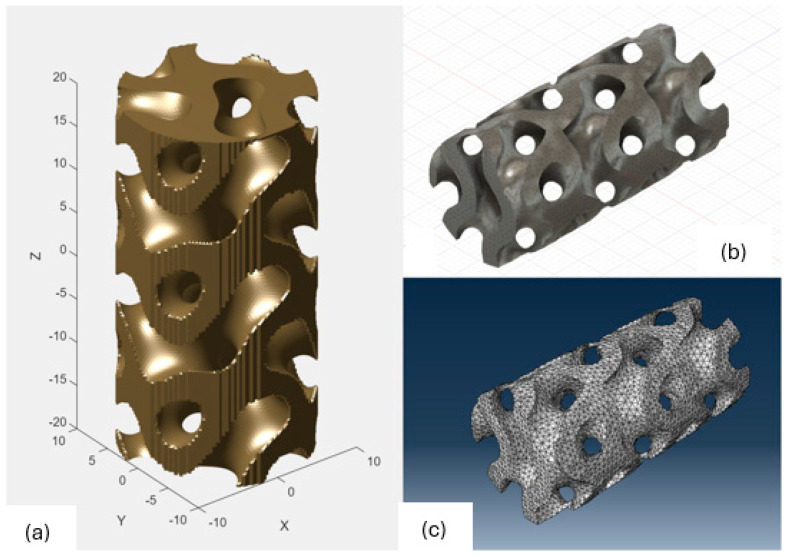
(**a**) Model in MSLattice. (**b**) Model repaired and mesh properties assigned in Fusion 360. (**c**) Model after being imported into Abaqus.

Samples models with different configurations (cell geometry, relative density, unit cell size) have been generated in MSLattice [23] software. This software generates STL files. Further, these model files were repaired in Fusion 360 software and mesh properties assigned. Models were imported into Abaqus software using “STL Import” plug-in. After that, geometry was created using “Create geometry from mesh” plug-in. Sample models at different stages of preparation are depicted in Figure 1.

### 2.4. Material Model

An elastic–perfectly plastic material model has been chosen for the study. This model is also used in the work by Peng et al. [8] and Di Frisco et al. [14]. In the work by Torres et al. [24], torsional properties of 3D-printed PLA specimens in static and cyclic loading are researched. Stress–strain curves for different PLA specimens show stress magnitudes, close to constant during the post-yield stage. Therefore, an elastic–perfectly plastic material model should give a good approximation of material behavior. The limitation of this material model is that data in the post-yield stage may not be accurate. Based on the literature review, the following material properties have been set: Young’s modulus E = 3500 MPa [24], Poisson’s ratio of 0.36 [14,24], yield stress of 60 MPa as an average value from different sources [14,24].

### 2.5. Load Condition Setup

**Figure 2:**
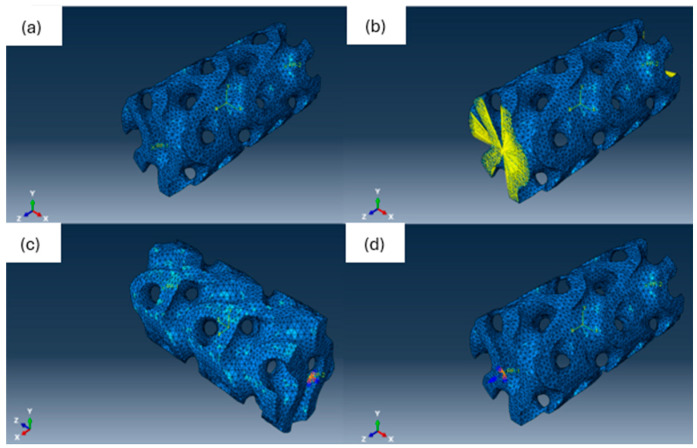
Load setup. (**a**) Reference points on model edges. (**b**) Linkage of the edge to the reference point. (**c**) Boundary conditions for right edge. Orange cones show translation restrictions; blue cones show rotation restrictions. (**d**) Restrictions and twist application on the right edge. Blue arrow shows the application of twist.

A displacement-driven simulation is conducted. This type of configuration is good for simulating the test on a torsion tester. First, reference points were created on each of the two edges of samples and linked to the edges. Then, boundary conditions and displacement have been applied. One edge of the sample was fixed in all translations and rotations. Another edge was fixed in translations, perpendicular to the sample axis. Translation along the sample axis was allowed. It was made to prevent tensile stresses from acting on a sample, which can interact with the final result. Rotations in the same plane with the sample axis have been restricted and twist was applied around the sample axis. The load setup is shown in Figure 2.

#### Postprocessing

After the simulation was completed, contour plots were obtained. Torque–Revolution curves were plotted using “XYData” option. To calculate shear stress–strain curves, the following formulas were used.

For shear stress:

(1) $$\tau =\frac{T\cdot r}{J}$$

where *τ* is shear stress, *T* is torque, *r* is sample radius, *J* is the polar moment of inertia. To calculate the polar moment of inertia, multiple sections of the sample were made, and their polar moment of inertia was calculated. The cross-section with minimal magnitude was considered critical for the whole structure, and used for the calculation, because according to Formula (1) in the cross-section, maximum shear stress will be present. A similar method for calculating the polar moment of inertia is utilized in the work by Hailu et al. [19,21].

For shear strain:

(2) $$\gamma =\frac{\theta \cdot r}{L}$$

where *γ* is shear strain, *θ* is twist angle, *r* is sample radius, *L* is sample length.

Shear modulus was calculated using the following formula:

(3) $$G=\;\frac{T\cdot L}{J\cdot \theta }$$

where *G* is shear modulus, *T* is torque, *L* is sample length, *J* is polar moment of inertia, *θ* is twist angle.

Shear yield stress was calculated at the intersection of the shear stress–strain curve with the line, plotted parallel to the linear part of the curve at an offset of 0.2% strain.

## 3. Results and Discussion

### 3.1. Von Mises Stress Distribution

The von Mises stress distribution in samples, obtained via simulation, is depicted in Table 3. Primitive samples with 20 mm unit cell size have the highest von Mises stress in the middle of unit cells. For the most samples, stress levels are lower in the center of samples. The only exclusion is Diamond samples with a unit cell size of 20 mm and relative densities of 30% and 50%, where high stress is also present near the sample axes. Also, high stress is concentrated. However, on the tips of these shapes, the stress is minimal, as these tips are not interconnected with other parts of the samples. For a relative density of 70%, stress is more redistributed to the edges. These protruding shapes become interconnected with each other and start taking stress. For unit cell size of 15 mm and 10 mm, the highest stress is present on the outer edges of the sample. For Gyroid, the highest stress is concentrated at the “grooves” inside the unit cells, which are close to the sample edge. For unit cell of 20 mm, high stress is also present at cell interconnections. For Primitive with unit cell size of 20 mm, there are distinct high-stress areas. The highest stress is present in the middle of unit cells. Also, high stress is present at cell interconnections. For unit cell size of 15 mm, high stress is only concentrated at cell interconnections. Due to these interconnections being smaller than for 20 mm unit cell size, the stress there is higher. In the middle of unit cells, stress is low. It is caused by material being added in the middle of unit cells, so that these regions are strengthened. For unit cell size of 10 mm, stress is distributed more evenly. High stress is also present at interconnections. In summary, it is noticeable that in Diamond and Gyroid, stress is distributed more evenly than in Primitive, which has distinct stress concentrations. However, with two or more unit cells in the direction perpendicular to the sample axis, yjr stress distribution becomes more even.

### 3.2. Effect of Cell Geometry

**Figure 3:**
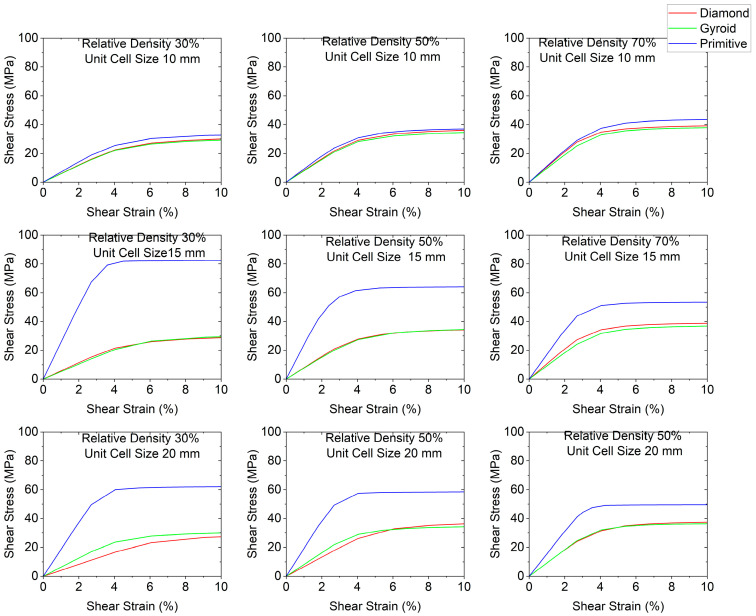
Stress–strain curves depending on cell geometry.

**Figure 4:**
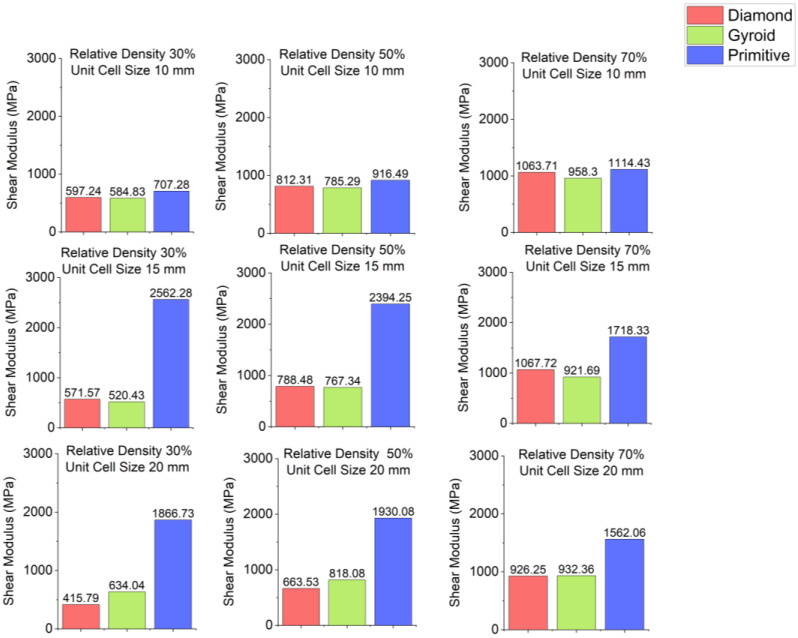
Shear modulus depending on cell geometry.

**Figure 5:**
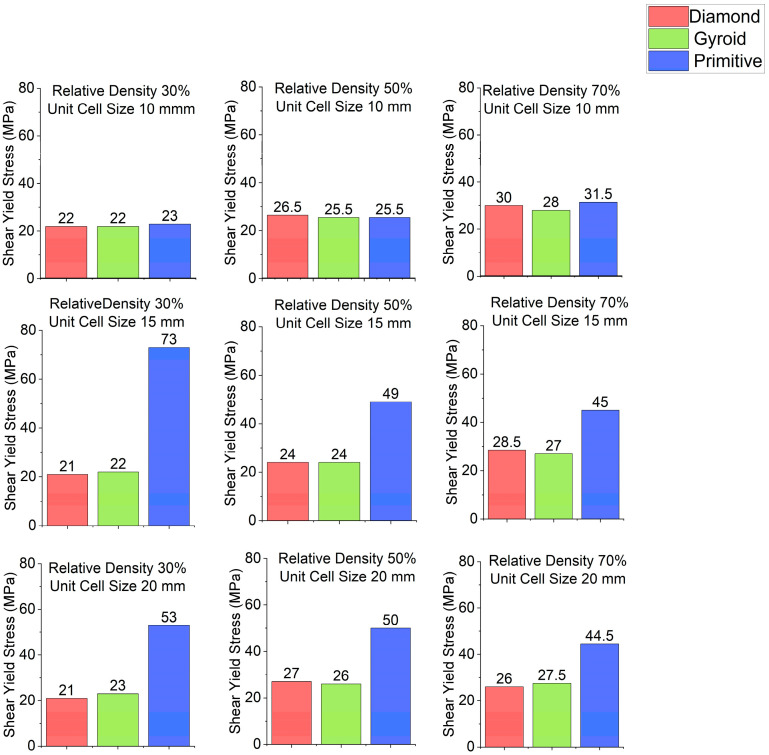
Shear yield stress depending on cell geometry.

Stress–strain curves for different cell geometries are plotted in Figure 3. Shear modulus charts are plotted in Figure 4 and shear yield stress charts are depicted in Figure 5. In all cases, Primitive has exhibited higher shear modulus than Gyroid and Diamond. Primitive exceeded in shear modulus. Shear yield stress was also the highest among all shapes, with the exclusion for unit cell size of 10 mm and relative density 50%, where Diamond with shear yield stress of 26.5 MPa has shown shear yield stress, slightly higher than Primitive and Gyroid, which had an equal stress values of 25.5 MPa. For Primitive with unit cell size of 10 mm, shear modulus and shear yield stress values were close to other geometries, but for unit cell sizes of 15 mm and 20 mm the values exceeded other cell geometries significantly; for different configurations Primitive had two to four times higher shear modulus. For the same unit cell sizes, Primitive has shown approximately two times higher shear yield stress. For a configuration with 30% relative density and 15 mm unit cell size, shear yield stress was almost four times larger than for Gyroid and Diamond. This trait can be explained by the fact that Primitive has a smaller cross-section than other cell geometries for the same relative density and unit cell size. Also, the Primitive cross-section consists of discrete shapes, while Gyroid and Diamond have continuous cross-sections. Also, this shape causes an uneven stress distribution in Primitive unit cell. Primitive has shown to withstand higher stress before yielding. Compared to other structures, for similar strain, Primitive had higher stress value. With the increase in the number of unit cells (decreased unit cell size), stress is distributed more evenly and values become close to other geometries. Regarding Diamond and Gyroid cell geometries, Diamond exceeds Gyroid in shear modulus in most cases except for 20 mm unit cell size and 30% and 50% relative density, where Gyroid has shown higher shear modulus. For relative density of 30%, Gyroid had shear modulus of 634 MPa against 416 MPa for Diamond. For the relative density of 50%, Gyroid has shown shear modulus of 818 MPa, while Diamond had 663.5 MPa. Regarding shear yield stress, Diamond and Gyroid have almost equal values with insignificant differences.

### 3.3. Effect of Relative Density

**Figure 6:**
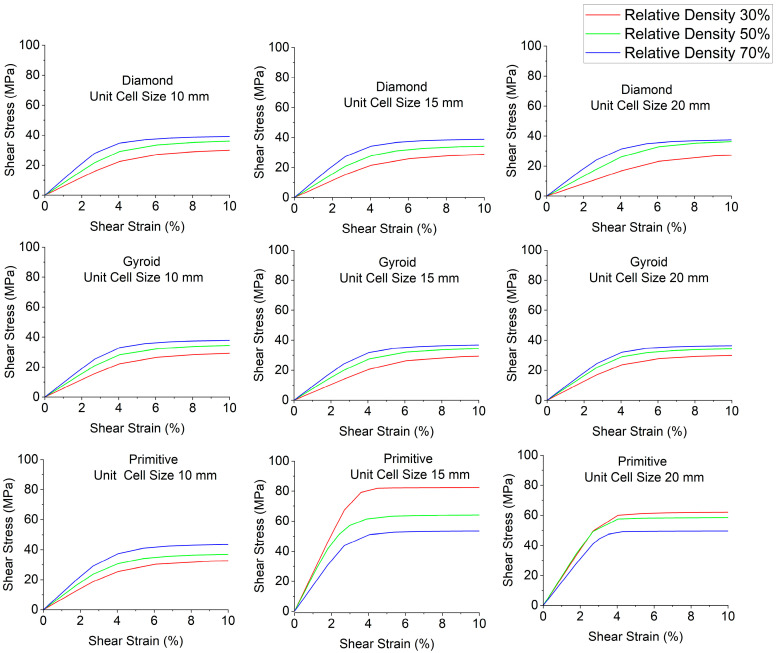
Stress–strain curves depending on relative density.

**Figure 7:**
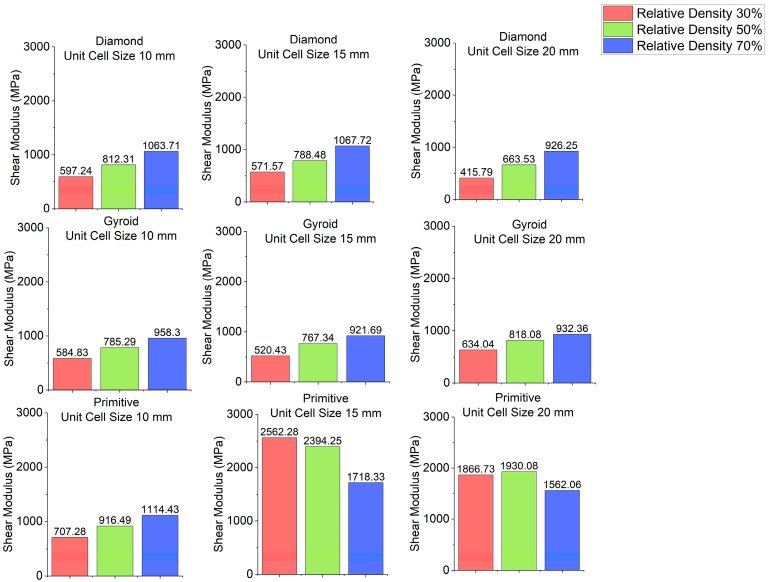
Shear modulus depending on relative density.

**Figure 8:**
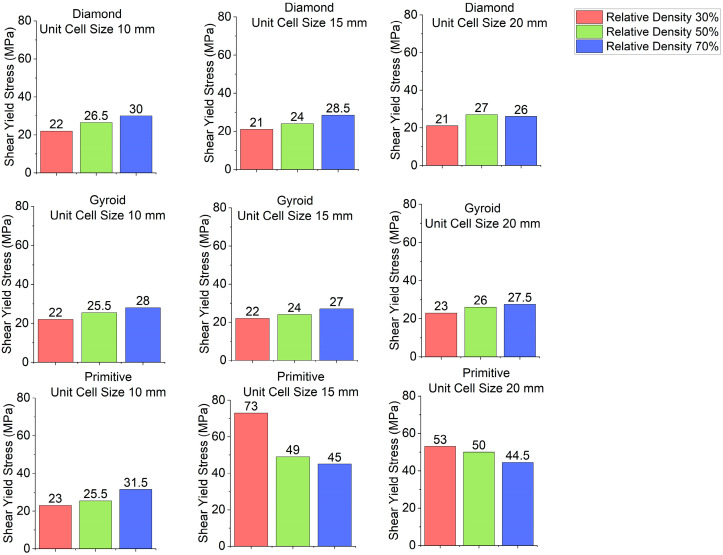
Shear yield stress depending on relative density.

Stress–strain curves depending on relative density are depicted in Figure 6; shear modulus charts are plotted in Figure 7 and shear yield stress charts are in Figure 8. Relative density has a strong influence on shear modulus and shear yield stress. Regarding shear modulus, for most cases, it increases with the increase in relative density. The exceptions to this trend are Primitive with unit cell size of 15 mm, where the opposite is observed: with the increase in relative density, shear modulus decreases. For Primitive with unit cell size of 20 mm, the sample with 50% relative density has slightly higher shear modulus than the one with 30% relative density, and the sample with 70% relative density has the lowest shear modulus. Regarding shear yield stress, for most cases the trend is the same: with the increase in relative density, shear yield stress increases, with the exception of Primitive with unit cell size of 20 mm and 15 mm, where shear yield stress decreases with the increase in relative density. Also, Diamond with unit cell size of 20 mm has a higher shear yield stress for the sample with 70% relative density, which has higher shear yield stress than the sample with 30% relative density, but slightly lower than the sample with 50% relative density.

### 3.4. Effect of Unit Cell Size

**Figure 9:**
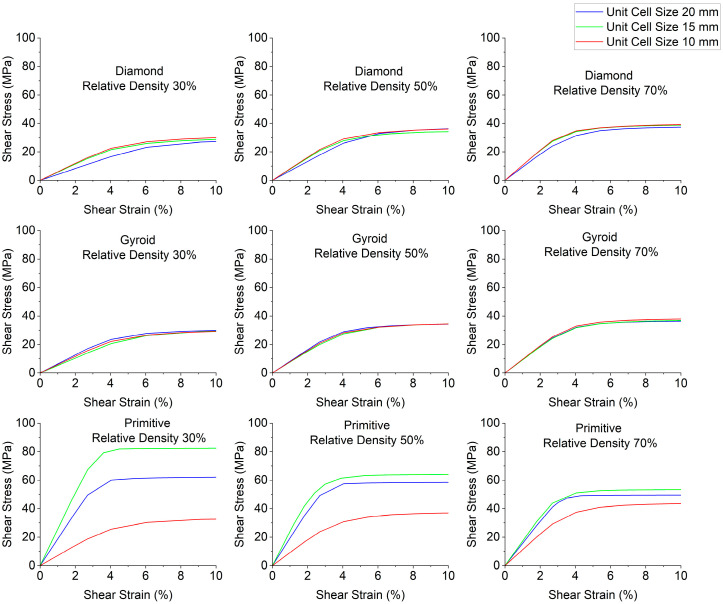
Stress–strain curves depending on unit cell size.

**Figure 10:**
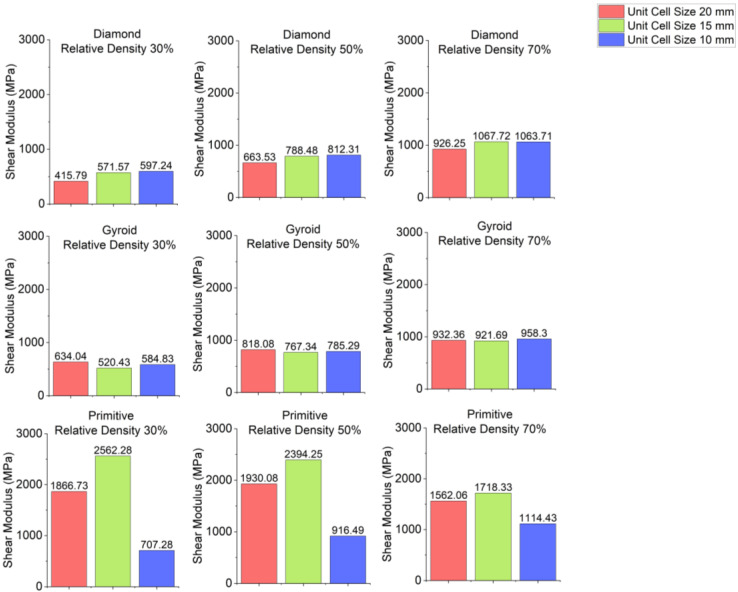
Shear modulus depending on unit cell size.

**Figure 11:**
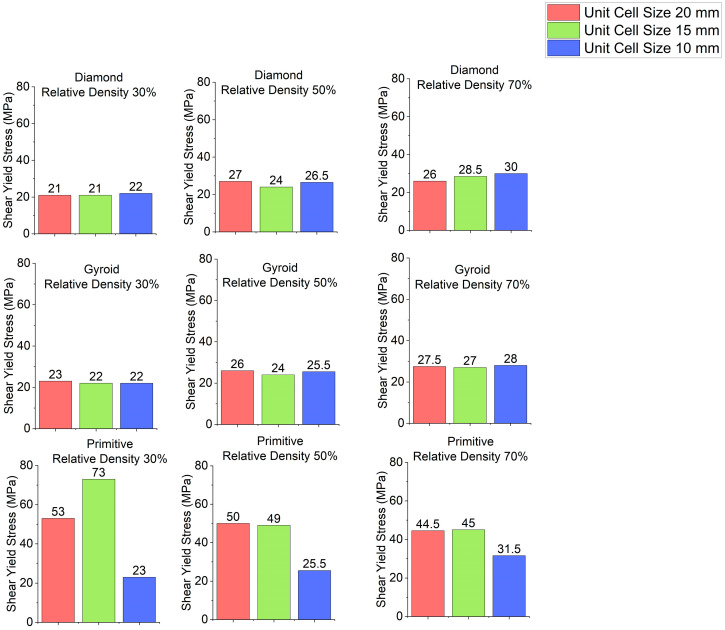
Shear yield stress depending on unit cell size.

The effect of unit cell size on stress–strain curves is shown in Figure 9. Charts showing the dependence of stress of shear modulus on unit cell size are depicted in Figure 10 and charts for shear yield stress are shown in Figure 11. For Diamond and Gyroid, unit cell size does not affect properties. Shear modulus and shear stress remain almost identical for different unit cell sizes. The exception is Primitive, where unit cell size has quite a significant influence. Regarding shear modulus, it is higher for the unit cell size of 15 mm than for 20 mm, but samples with unit cell size of 10 mm have significantly lower shear modulus. Regarding shear yield stress, for Primitive with relative density of 30%, the highest value is for the unit cell size of 15 mm, followed by the sample with 20 mm unit cell size and 10 mm unit cell size. For Primitive with 50% and 70% relative density, shear yield strength is almost identical for the samples with 20 mm and 15 mm unit cell size. Samples with 10 mm unit cell size have significantly lower shear yield stress. The reason for such difference in properties for Primitive cell geometry is that its cross-section is discrete, and stress distribution is uneven. However, this can be improved by increasing the number of unit cells.

### 3.5. Comparison with Results in Reviewed Literature

The effect of relative density on torsion properties of samples is studied in the works by Yánez et al. [16,17], as well as in the work by Timercan et al. [18]. Yánez et al. [16] study torsional properties of several skeletal Gyroid shapes, with porosity levels of 75% and 90%. From the results of their work, it is noticed that with the increase in porosity level (decrease in relative density), shear modulus and shear yield strength decrease. For Gyroid, the same result is obtained in the current work. For sheet gyroids, porosity levels of 65%, 70% and 80% are studied. Sheet gyroids presented are normal and with drilled holes. For normal Gyroid, with the increase in porosity level, shear modulus and shear yield stress decrease. For drilled Gyroid, shear yield stress also decreases with the increase in porosity level, shear modulus is higher for 70% porosity level than for 80%, but for 65% shear modulus is slightly lower, compared to 70%. For normal sheet Gyroid, results are consistent with the current work. In the work by Timercan et al. [18], sheet Gyroid and skeletal Diamond shapes are studied. For both shapes, shear modulus and shear yield stress decrease with the increase in porosity. This coincides with results obtained in the current work for Gyroid and most configurations of Diamond, with the exception of Diamond with 20 mm unit cell size. In this case, shear yield stress for 70% relative density was lower than the value for 50% relative density. Regarding the effect of cell geometry, results are different from the current work, but in the study by Timercan et al., skeletal Diamond is studied, while in this work, sheet Diamond is studied, so the comparison regarding the effect of cell geometry is not representative. In the study by Hailu et al. [21] Gyroid, Primitive TPMS structures are studied, as well as Vertical-Inclined strut-based structure. As a result, Gyroid had higher shear modulus than Primitive, which contradicts the results of the current study. Primitive has resulted in higher shear modulus than Gyroid. In the work by Jijun Jiang et al. [22], torsion properties of column-based Diamond, Gyroid, IWP and Primitive structures are researched. Von Mises stress distribution obtained in their work is similar to the distribution resulting from the current study. Regarding stress–strain curves, shear modulus and shear yield stress, Primitive had the lowest shear modulus and shear yield stress, while Gyroid had the highest values. Diamond and IWP had almost identical values between Primitive and Gyroid. These results are different from the results obtained in the current work; Primitive has the highest shear modulus and shear yield stress, and Gyroid and Diamond have significantly lower values, Gyroid slightly higher than Diamond. The effect of unit cell size is studied by Naghavi et al. [25]. With the increase in unit cell size, shear modulus and shear yield stress decrease, so with the varying unit cell size, properties are not constant. However, in the work, sheet thickness is kept constant, and pore size is changed. This design of experiment is different from the current work. Also, in the design of experiment by Naghavi et al. [25], porosity level is also changed with the change in unit cell size.

## 4. Conclusions

In this work, torsion properties of Diamond, Gyroid and Primitive TPMS structures are researched, depending on their relative density and unit cell size, using the finite element method. Twenty-seven simulations have been conducted.

- Shear modulus and shear yield stress have been calculated from the curves.
- Regarding the effect of cell geometry, Primitive with unit cell sizes of 15 mm and 20 mm had significantly higher shear modulus of 1500–2500 MPa depending on the condition, against 400–1000 MPa for Diamond and Gyroid samples. Shear yield stress was also significantly higher at 44–73 MPa, while Diamond and Gyroid have values of 21–27 MPa. For unit cell size of 10 mm, shear modulus and shear stress had close values across geometries, with shear modulus values of 500–1100 MPa and shear yield stress of 22–31 MPa, depending on the condition.
- Effect of relative density had a significant influence on all the samples. For Diamond and Gyroid, as well as for Primitive with unit cell size 10 mm, with the increase in relative density, shear modulus increased from 400–600 MPa for 30% relative density, up to 900–100 MPa for 70% relative density. Shear yield stress has also increased for higher relative density from 21–23 MPa to 27–31 MPa. For Diamond with unit cell size of 20 mm, the sample with relative density 50% had slightly higher shear yield stress of 27 MPa than the sample with 70% relative density with 26 MPa shear yield stress. For Primitive with 15 mm and 20 mm unit cell, the effect of relative density was the opposite: with the increase in relative density, shear modulus and shear yield stress decreased.
- Regarding the effect of unit cell size, Diamond and Gyroid were not affected; with the change of the unit cell size, shear modulus and shear yield stress values were about the same. For Primitive, however, unit cell size had a significant influence. Primitive samples with 15 mm unit cell size had the highest shear modulus of 1700–2500 MPa, depending on the configuration, whereas samples with 20 mm unit cell size had the lowest shear modulus of 700–1100 MPa. Regarding shear yield stress of Primitive samples, it was almost the same for samples with 10 mm and 15 mm unit cell size, with values of 45–50 MPa, with the exception of the sample with unit cell size 15 mm and relative density of 30%, which had a shear yield stress value of 73 MPa. Samples with 20 mm unit cell size had the lowest shear yield stress of 23–31 MPa.

## Figures and Tables

**Table 1:** Tested conditions of TPMS samples.

	Diamond		
	Unit Cell Size 10 mm	Unit Cell Size 15 mm	Unit Cell Size 20 mm
Relative Density 30%	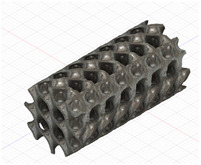	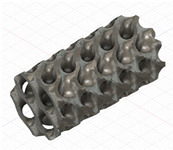	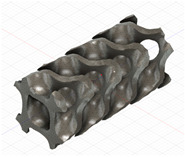
Relative Density 50%	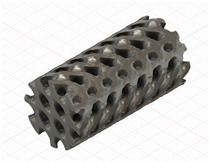	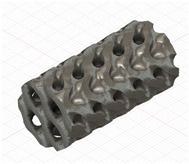	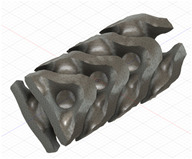
Relative Density 70%	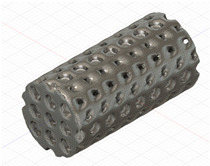	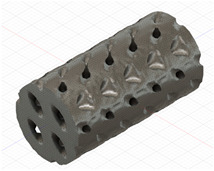	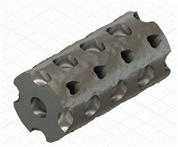
	Gyroid		
	Unit Cell Size 10 mm	Unit Cell Size 15 mm	Unit Cell Size 20 mm
Relative Density 30%	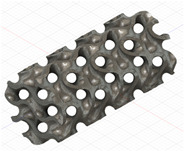	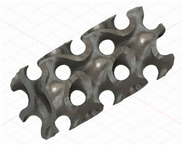	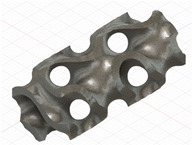
Relative Density 50%	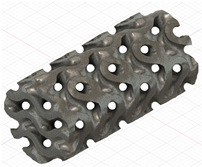	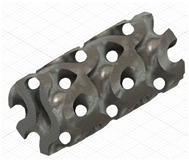	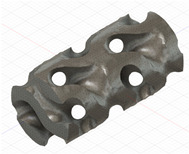
Relative Density 70%	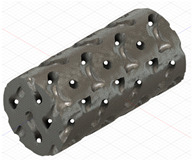	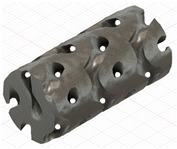	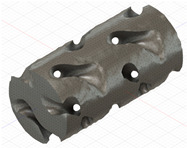
	Primitive		
	Unit Cell Size 10 mm	Unit Cell Size 15 mm	Unit Cell Size 20 mm
Relative Density 30%	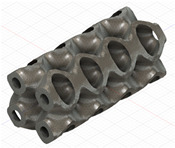	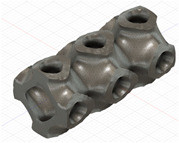	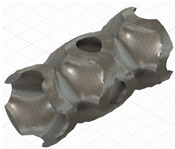
Relative Density 50%	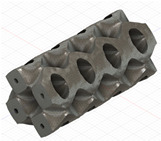	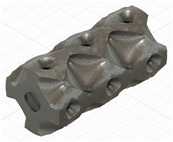	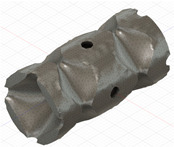
Relative Density 70%	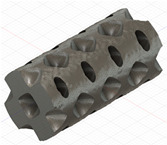	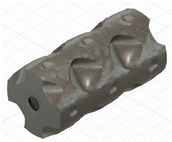	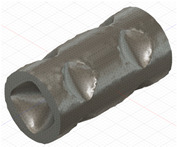

**Table 2:** Error in maximum reaction moment between mesh densities in %.

Compared Mesh Densities	Unit Cell Size, mm		
	10 mm	15 mm	20 mm
45 to 30	25.05%	0.71%	3.83%
60 to 45	0.90%	0.62%	0.44%
75 to 60	0.03%	0.36%	0.23%

**Table 3:** Von Mises stress distribution in samples.

	Diamond			
	Unit Cell Size 10 mm	Unit Cell Size 15 mm	Unit Cell Size 20 mm	Spectrum (MPa)
Relative density 30%	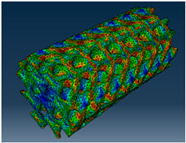	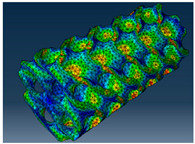	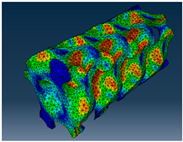	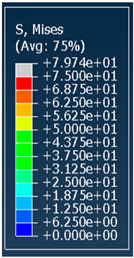
Relative density 50%	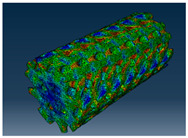	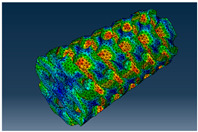	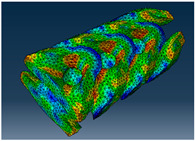	
Relative density 70%	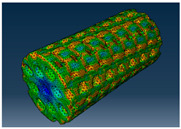	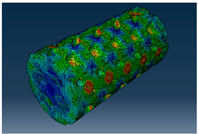	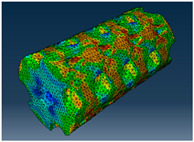	
	Gyroid			
	Unit Cell Size 10 mm	Unit Cell Size 15 mm	Unit Cell Size 20 mm	Spectrum (MPa)
Relative density 30%	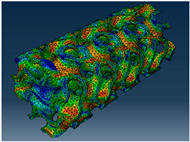	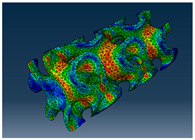	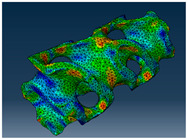	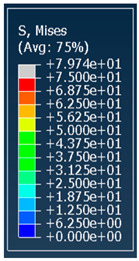
Relative density 50%	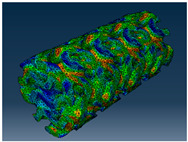	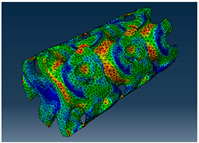	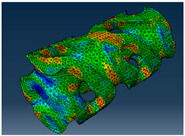	
Relative density 70%	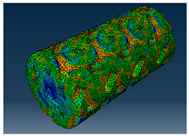	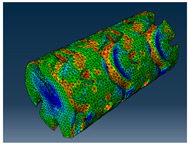	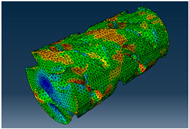	
	Primitive			
	Unit Cell Size 10 mm	Unit Cell Size 15 mm	Unit Cell Size 20 mm	Spectrum (MPa)
Relative density 30%	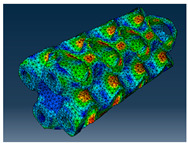	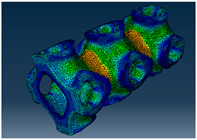	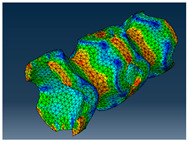	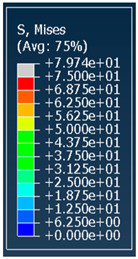
Relative density 50%	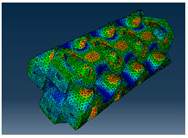	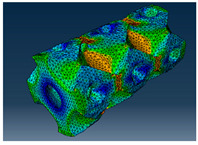	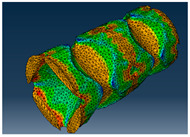	
Relative density 70%	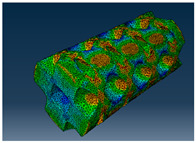	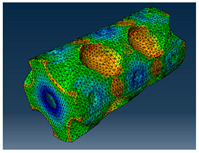	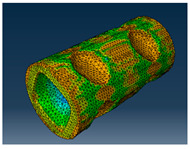	

## Data Availability

Data can be made available upon request to the corresponding author.
